# Genome-Wide Identification of DNA Methyltransferase and Demethylase in *Populus* sect. Turanga and Their Potential Roles in Heteromorphic Leaf Development in *Populus euphratica*

**DOI:** 10.3390/plants14152370

**Published:** 2025-08-01

**Authors:** Chen Qiu, Jianhao Sun, Mingyu Jia, Xiaoli Han, Jia Song, Zhongshuai Gai, Zhijun Li

**Affiliations:** 1 Xinjiang Production & Construction Corps Key Laboratory of Protection and Utilization of Biological Resources in Tarim Basin, Alar 843300, China; qiuchentea@163.com (C.Q.); sunjianhaotea@163.com (J.S.); jiamingyu0717@163.com (M.J.); lilyan0509@163.com (X.H.); nxy@163.com (J.S.); 2 College of Life Science and Technology, Tarim University, Alar 843300, China; 3 Desert Poplar Research Center, Tarim University, Alar 843300, China

**Keywords:** DMT gene famliy, DML gene family, *Populus euphratica*, *Populus pruinosa*, heteromorphic leaf

## Abstract

DNA methylation, mediated by DNA methyltransferases (DMTs) and demethylases (DMLs), is an important epigenetic modification that maintains genomic stability and regulates gene expression in plant growth, development, and stress responses. However, a comprehensive characterization of these gene families in *Populus* sect. Turanga remains lacking. In this study, eight *PeDMT* and two *PeDML* genes were identified in *Populus euphratica*, and six *PpDMT* and three *PpDML* genes in *Populus pruinosa*. Phylogenetic analysis revealed that DMTs and DMLs could be classified into four and three subfamilies, respectively. The analysis of cis-acting elements indicated that the promoter regions of both DMTs and DMLs were enriched with elements responsive to growth and development, light, phytohormones, and stress. Collinearity analysis detected three segmentally duplicated gene pairs (*PeDMT5/8*, *PeDML1/2*, and *PpDML2/3*), suggesting potential functional diversification. Transcriptome profiling showed that several *PeDMTs* and *PeDMLs* exhibited leaf shape- and developmental stage-specific expression patterns, with *PeDML1* highly expressed during early stages and in broad-ovate leaves. Whole-genome bisulfite sequencing revealed corresponding decreases in DNA methylation levels, suggesting that active demethylation may contribute to heteromorphic leaf formation. Overall, this study provides significant insights for exploring the functions and expression regulation of plant DMTs and DMLs and will contribute to future research unraveling the molecular mechanisms of epigenetic regulation in *P. euphratica*.

## 1. Introduction

DNA methylation is a conserved epigenetic gene-regulation mechanism that involves the addition of a methyl group to the 5′ position of cytosine residues [1]. This modification plays a central role in the regulation of gene expression, genome stability, and developmental processes in both animals and plants [2,3]. Unlike animals, where methylation predominantly occurs in the CG context [4], plant genomes exhibit methylation in three sequence contexts: CG, CHG, and CHH (where H represents A, T, or C) [5,6]. The establishment, maintenance, and removal of these methylation marks are mediated by a coordinated set of enzymes, including DNA methyltransferases (DMTs) and demethylases (DMLs), enabling dynamic epigenetic regulation in response to developmental and environmental cues [5,7].

In plants, DMTs are classified into four major families based on their domain structures and biological functions: methyltransferase (MET), chromomethylase (CMT), domains rearranged methylase (DRM), and DNA methyltransferase 2 (DNMT2) [8]. MET1 primarily maintains CG methylation during DNA replication [9], while CMT2 and CMT3 are involved in maintaining CHH and CHG methylation [10], often in conjunction with histone modifications [11]. DRM2 functions as the main de novo methyltransferase, particularly in the RNA-directed DNA methylation (RdDM) pathway, which utilizes 24 nt small interfering RNAs to target methylation across all sequence contexts [12]. In contrast, active DNA demethylation is mediated by repressor of silence 1 (ROS1), transcriptional activator demeter (DME), demeter-like protein 2 (DML2), and demeter-like protein 3 (DML3) [13], which initiate base excision repair pathways to remove 5-methylcytosine, thus counteracting excessive or unwanted methylation.

In recent years, accumulating evidence has demonstrated that DMTs and DMLs, as the key enzymes regulating genome-wide DNA methylation levels, not only play important regulatory roles in plant responses to various abiotic stresses, including cold [14], drought [15], heat [16], and salt [17,18], but are also widely involved in multiple stages of plant growth and development. For instance, during orange fruit ripening, a global increase in DNA methylation was associated with reduced expression of *CsDML* genes [19]. Similarly, in tomato, *SlDML2* precisely regulated fruit ripening by specifically removing methylation marks in the promoter regions of ripening-related genes [20]. In rice, the DML gene *OsROS1* modulated endosperm development through active DNA demethylation, influencing nutrient composition of the grain [21], while loss-of-function mutants of the DMT genes *OsCMT3* and *OsDRM2* exhibited early flowering, dwarfism, and severe developmental defects [22,23]. Further supporting this, studies in *Arabidopsis thaliana* have shown that mutations in DMT genes such as *AtDRM1*, *AtDRM2*, and *AtCMT3* resulted in altered leaf morphology, impaired reproductive development, and growth abnormalities [24].

Despite these advances, most functional studies of DMT and DML genes have focused on model species or crops [15,25,26], whereas their roles in woody plants, especially those adapted to extreme environments, remain poorly understood [27,28]. *Populus euphratica* Oliv. and *Populus pruinosa* Schrenk are typical desert heterophyllous species in *Populus*, with four and three distinct leaf types, respectively [29,30,31]. Both species are widely distributed across the arid regions of northwest China and Central Asia, exhibiting pronounced phenotypic plasticity associated with different developmental stages. These leaves are not only morphologically diverse but also functionally differentiated: previous studies have shown that broad leaves exhibit stronger drought tolerance than narrow leaves, including traits such as thicker palisade tissues and higher photosynthetic activity [32,33,34,35]. Such differentiation highlights their ecological plasticity and suggests a regulatory link between leaf morphology and environmental adaptation. Therefore, these heteromorphic leaves offer a valuable in vivo system for investigating the epigenetic control of developmental plasticity. Based on whole-genome data, this study systematically identified members of the DMT and DML gene families in both species, analyzing their phylogenetic relationships, gene structures, conserved domains, and chromosomal distributions. Although *P. euphratica* and *P. pruinosa* share similarities in morphology and ecological adaptation, *P. euphratica* has a wider distribution, stronger stress tolerance, and more pronounced phenotypic diversity [36,37,38]. Therefore, a more in-depth analysis of the transcriptome and DNA methylation expression patterns of *P. euphratica* was conducted to explore the potential regulatory roles of *PeDMT* and *PeDML* in heterophyllous leaf formation and adaptive evolution. This study lays the groundwork for future functional analyses of DMT and DML genes in *Populus* species, while also advancing our understanding of their epigenetic roles in regulating heteromorphic leaf development and adaptation to arid environments.

## 2. Results

### 2.1. Identification and Chromosomal Classification of DMTs and DMLs

**Figure 1:**
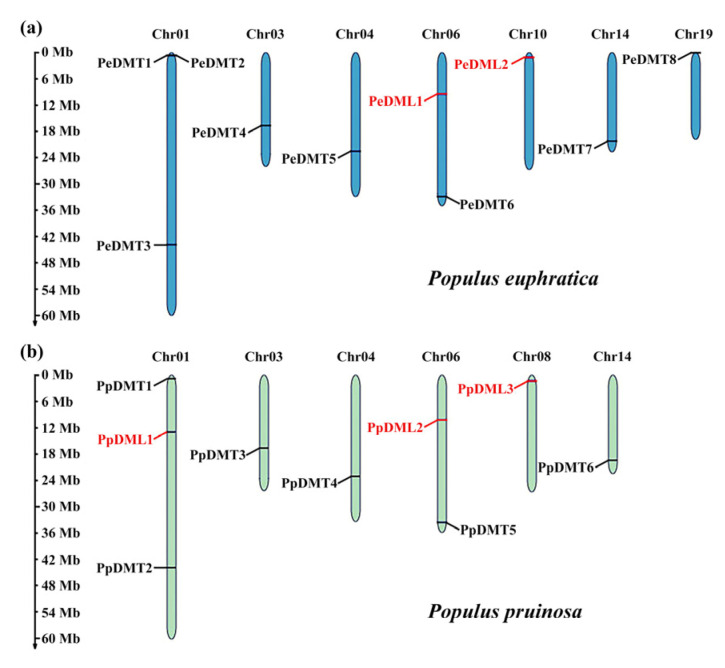
Genomic distribution of DMTs and DMLs across chromosomes of (**a**) *P. euphratica* and (**b**) *P. pruinosa*.

Based on Hidden Markov Model (HMM) profiling and BLASTP homology searches, a total of eight *PeDMTs* (*PeDMT1* to *PeDMT8*) and two *PeDMLs* (*PeDML1* to *PeDML2*) genes were identified in *P. euphratica*, while six *PpDMTs* (*PpDMT1* to *PpDMT6*) and three *PpDMLs* (*PpDML1* to *PpDML3*) genes were identified in *P. pruinosa*. All genes were sequentially numbered according to their physical positions from top to bottom on Chr01-19. In *P. euphratica*, 10 genes were distributed across seven chromosomes, with Chr01 harboring the highest density. In *P. pruinosa*, nine genes were located on six chromosomes, also with the highest density observed on Chr01 (Figure 1a). Although the localization of DMT and DML homologous is highly conserved between the two species, interspecific differences in gene numbers and chromosomal distributions—such as DMT-specific enrichment on Chr19 in *P. euphratica* and DML-specific enrichment on Chr01 in *P. pruinosa*—likely reflect functional differentiation during adaptive evolution.

The physicochemical characteristics of the DMT and DML family proteins in *P. euphratica* and *P. pruinosa* were systematically analyzed (Table S1). Protein lengths exhibited substantial variation across gene families, with DML members consistently displaying significantly longer amino acid sequences compared to DMT proteins. Specifically, DMT proteins ranged from 66 [PeDMT1, molecular weight (MW): 7.5 kDa] to 381 amino acids (PpDMT1, MW: 43.6 kDa), while DML proteins spanned 862 (PeDML1, MW: 96.9 kDa) to 1876 amino acids (PpDML3, MW: 210.0 kDa). The isoelectric point (pI) ranged from 5.16 (PeDMT7 and PpDMT6) to 9.22 (PeDMT5 and PpDMT4). Notably, 57% of DMT members and 40% of DML members were acidic (pI < 7.0). The analysis of protein instability indices revealed that 64% of DMT proteins (instability index < 40) were stable, whereas all DML proteins (instability index > 40) were unstable. Hydrophobicity analysis, assessed using grand average of hydrophobicity (GRAVY) values, revealed that the majority of DMT proteins exhibited hydrophilic properties (GRAVY < 0), with the exception of PeDMT2, PeDMT7, and PpDMT6, which displayed hydrophobic characteristics (GRAVY > 0). In contrast, all DML proteins showed consistently hydrophilic profiles, with GRAVY values ranging from −0.754 (PpDML1) to −0.58 (PeDML1). These variations underscore structural and functional divergence between the DMT and DML families, potentially reflecting adaptive specialization in *Populus* sect. Turanga species.

### 2.2. Protein Structure and Subcellular Localization Prediction

According to secondary structure predictions (Table S2), all proteins consisted of three typical structural elements: alpha helix, extended strand, and random coil. Among them, the proportion of random coil was the highest, ranging from 47.98% to 71.38%, followed by alpha helix, whereas extended strand accounted for the lowest proportion. The three-dimensional structures of 10 DMT proteins from *P. euphratica* and *P. pruinosa* were successfully predicted using the SWISS-MODEL online server (Figure S1). The modeling results indicated that the GMQE values of all these proteins exceeded 0.7, suggesting a high reliability of the predictions. Additionally, subcellular localization predictions using WOLF PSORT (Table S3) showed that all DML members were localized to the cell nucleus, suggesting their potential involvement in nuclear DNA demethylation processes. In contrast, DMT proteins displayed more diverse subcellular localization patterns, including the nucleus, cytoplasm, and chloroplast.

### 2.3. Phylogenetic Relationship of DMTs and DMLs

**Figure 2:**
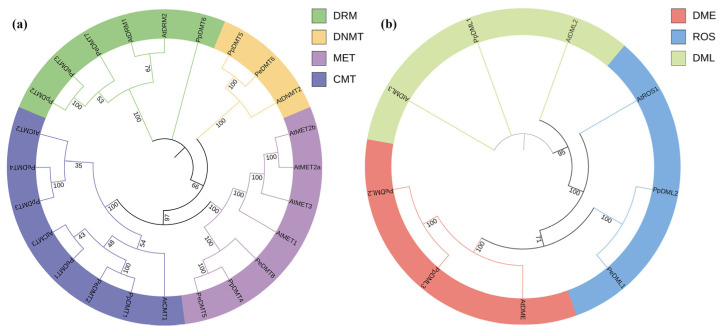
Phylogenetic tree of (**a**) DMT and (**b**) DML gene family among *A. thaliana*, *P. euphratica*, and *P. pruinosa*. Branch support was assessed using bootstrap values based on 1000 replicates.

To elucidate the phylogenetic relationships of DMTs and DMLs in plants, 24 DMT and 9 DML protein sequences from *A. thaliana*, *P. euphratica*, and *P. pruinosa* were aligned and used to construct phylogenetic trees (Figure 2). The DMT family was classified into four major categories: DRM, DNMT, MET, and CMT (Figure 2a), while the DML family clustered into three distinct groups: DME, ROS, and DML (Figure 2b). Phylogenetic analysis revealed closer evolutionary relationships between MET and CMT groups, as well as between ROS and DME groups. Notably, a DML gene was present in *P. pruinosa* but absent in *P. euphratica*, which may indicate species-specific variation in gene family composition. However, whether this difference is related to adaptive evolution requires further functional validation.

### 2.4. Conserved Motifs, Protein Domains, and Gene Structures of DMTs and DMLs

**Figure 3:**
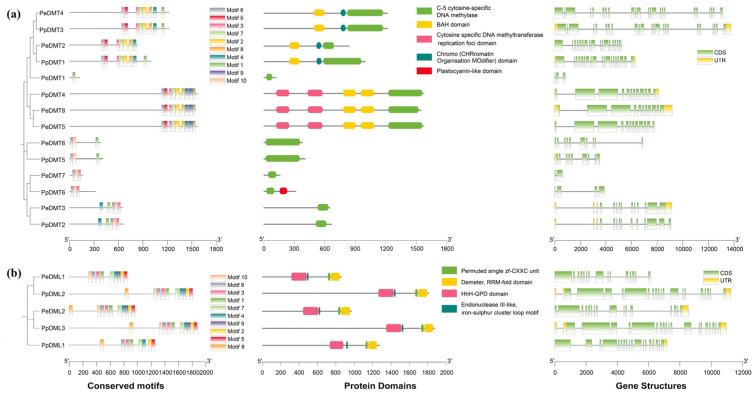
Analysis of phylogenetic relationships, conserved motifs, gene structure, and protein domains among (**a**) DMTs and (**b**) DMLs in *P. euphratica* and *P. pruniosa*.

The phylogenetic trees of the DMT and DML gene families from *P. euphratica* and *P. pruniosa* were constructed using MEGA12 (v12.0.11) software, followed by a comprehensive characterization of their conserved motifs, protein domains, and gene structures (Figure 3a,b). Through MEME analysis, 10 motifs (designated Motif 1–Motif 10) were identified across 14 DMT and 5 DML genes, respectively (Table S4). Significant divergence in motif conservation patterns was observed: Motif 1, Motif 3, and Motif 6 dominated in DMT members, whereas Motif 1–Motif 7 were highly conserved in DML members. All DMT family members contained the C-5 cytosine-specific DNA methylase domain, while all DML family members contained the permuted single zf-CXXC unit, RRM-fold, and HhH-GPD domains. Structural analysis revealed that all 19 genes possessed multiple exons and introns, with phylogenetically clustered genes exhibiting similar exon numbers and arrangement patterns. Notably, DMT genes displayed substantial length variation (600 bp to 13,761 bp) with 2–23 exons, while DML genes maintained relatively stable lengths (6113 bp to 11,268 bp) and conserved exon numbers (15–19 exons). These findings demonstrated that DMT and DML, as independently evolved gene families, retain structural conservation within their respective families while achieving functional specificity through adaptive divergence in key architectural features, including exon–intron organization and motif composition.

### 2.5. Interspecific and Intraspecific Collinearity Analysis of DMTs and DMLs Among Three Species

**Figure 4:**
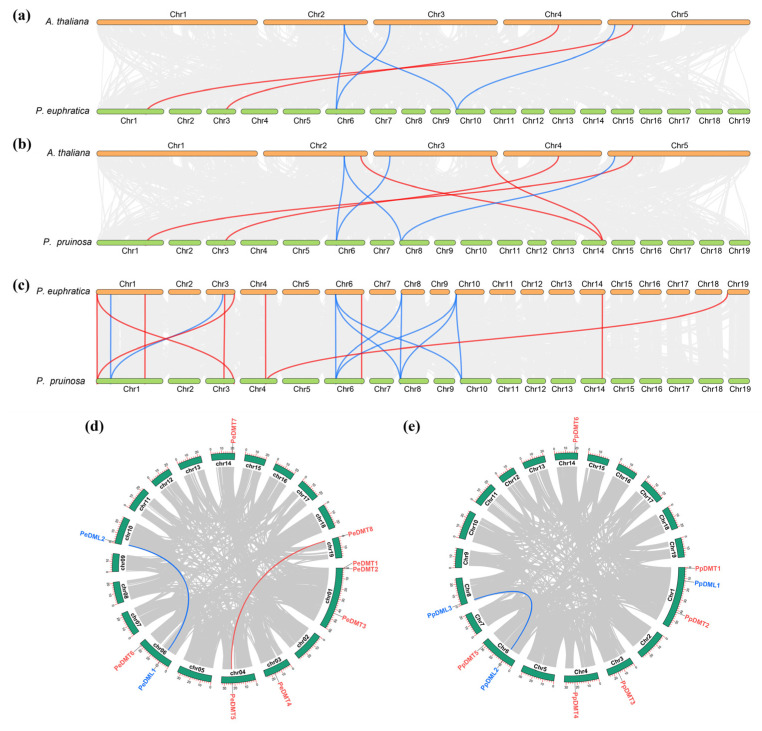
Collinearity analysis of DMTs and DMLs gene family. (**a**) *P. euphratica* vs. *A. thaliana*, (**b**) *P. pruinosa* vs. *A. thaliana*, (**c**) *P. euphratica* vs. *P. pruinosa*, (**d**) intraspecific collinearity in *P. euphratica*, (**e**) intraspecific collinearity in *P. pruinosa*. The red lines highlight collinear DMT pairs, while the blue lines highlight collinear DML pairs.

To elucidate the evolutionary dynamics of DMT and DML gene families, a comprehensive comparative genomic analysis was conducted among *P. euphratica*, *P. pruinosa*, and *A. thaliana*. The collinearity analysis revealed distinct conservation patterns: for DMT genes, two, four, and nine collinear pairs were identified between *P. euphratica*–*A. thaliana*, *P. pruinose*–*A. thaliana*, and *P. euphratica*–*P. pruinosa*, respectively (Figure 4a–c). Similarly, DML genes exhibited four collinear pairs in both *P. euphratica*–*A. thaliana* and *P. pruinose*–*A. thaliana* comparisons, whereas ten pairs were observed between the two *Populus* species, indicating higher conservation. Notably, genome-wide screening detected two segmentally duplicated gene pairs (*PeDMT5*/*PeDMT8* and *PeDML1*/*PeDML2*) in *P. euphratica* (Figure 4d), contrasting with only one conserved pair (*PpDML2*/*PpDML3*) in *P. pruinosa* (Figure 4e). These findings demonstrated that, despite the evolutionary divergence among different plant species, the DMT and DML gene families have exhibited a degree of conservation during the evolutionary process. Moreover, the differential retention of duplicated gene copies between the two closely related *Populus* species suggests lineage-specific evolutionary trajectories following segmental duplication events.

### 2.6. Analysis of the Cis-Acting Elements in the Promoter Region of DMTs and DMLs

**Figure 5:**
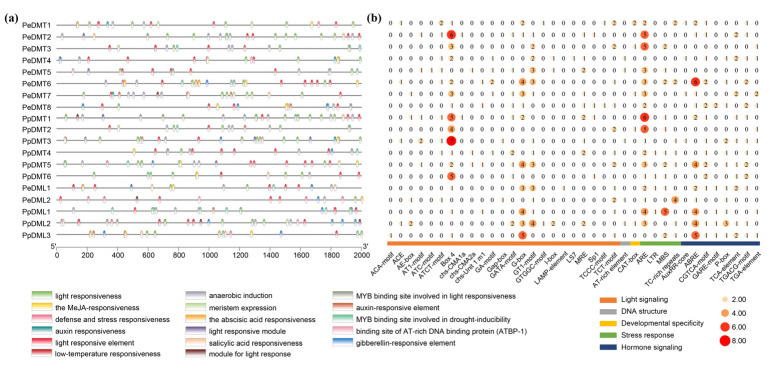
Analysis of cis-acting elements in *DMT* and *DML*. (**a**) Predicted cis-acting elements in the 2000 bp upstream regions of DMT and DML genes. (**b**) The abundance of cis-acting elements in each gene.

To elucidate the potential regulatory roles of *DMT* and *DML* genes in *P. euphratica* and *P. pruinosa*, the cis-acting regulatory elements within the 2 kb upstream regions of 19 DNA methylation-related genes were analyzed using the PlantCARE database (Figure 5a). The results revealed that both gene families contain a large number of cis-acting elements, which can be broadly categorized into five groups: light signaling, developmental specificity, hormone signaling, stress response, and DNA structure-related elements. Among these, light-signaling elements were the most abundant and widely distributed. Notably, the light-signaling elements G-box, Box-4, and GT1-motif, the stress-responsive element ARE, and the hormone-signaling element ABRE were commonly present in most *DMT* and *DML* genes (Figure 5b), suggesting that the *DMT* and *DML* gene families play important roles in plant growth, development, and environmental responses.

### 2.7. Expression Pattern of PeDMTs and PeDMLs During the Development of Heteromorphic Leaves of P. euphratica

**Figure 6:**
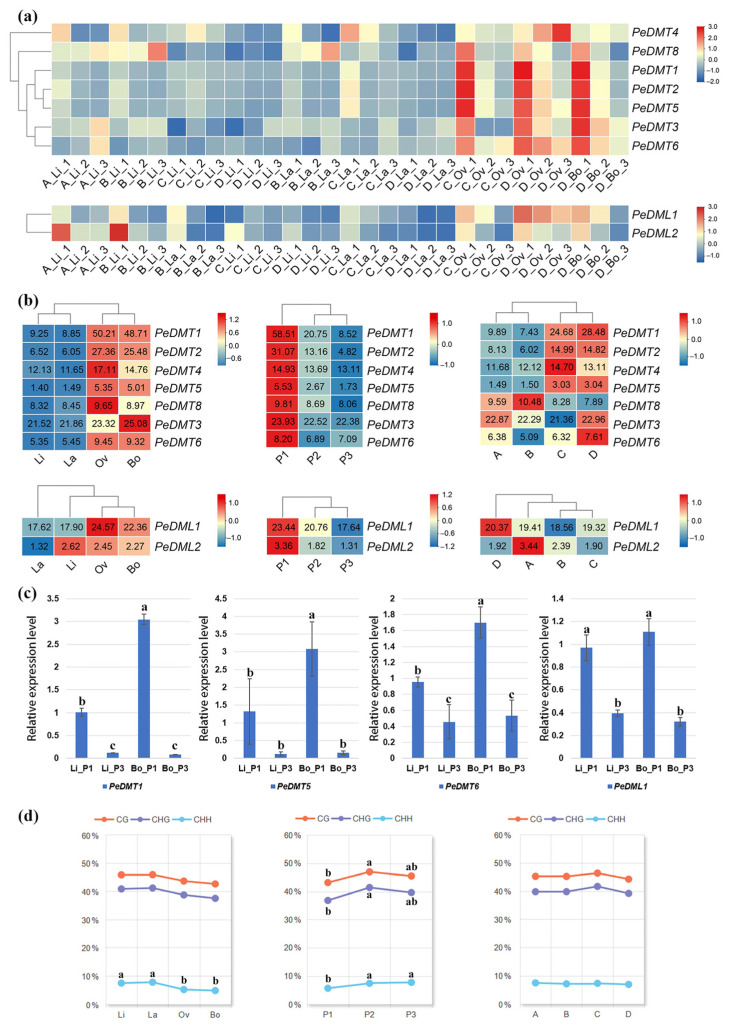
Expression of *PeDMT* and *PeDML* genes in RNA-seq and relative expression level. Heatmaps showing relative expression levels of seven *PeDMT* and *PeDML* genes in (**a**) 30 groups and in (**b**) the different comparison groups. (**c**) The relative expression level of *PeDMT1*, *PeDMT5*, *PeDMT6*, and *PeDML1* in 4 samples. (**d**) The DNA methylation rates in the different comparison groups. Tree stages: A Stage (3 years): only Li; B Stage (5 years): Li and La; C Stage (7 years): Li, La, and Ov; D Stage (8 years): Li, La, Ov, and Bo. Leaf stages: P1: complete leaf flattening; P2: intermediate stage; P3: maturation phase. Significant differences were determined by ANOVA with the 0.05 *p*-value threshold. Comparison letters were shown only when differences were statistically significant.

To investigate the functions of DMT and DML genes in the development of heteromorphic leaves in *P. euphratica*, this study systematically analyzed the expression patterns of these two gene families across four heteromorphic leaf types [linear (Li), lanceolate (La), ovate (Ov), and broad-ovate (Bo)] during their developmental stages (P1–P3, A–D). Among the DMT genes, except for *PeDMT7* (PeuTF14G01466), which showed negligible expression [transcripts per million (TPM) < 1] across all samples, the other DMT genes exhibited stage-specific expression patterns in both narrow leaves (Li and La) and broad leaves (Ov and Bo). Specifically, *PeDMT1*, *PeDMT2*, *PeDMT3*, *PeDMT4*, *PeDMT5*, *PeDMT6*, and *PeDMT8* showed dominant expression in broad leaves (Ov and Bo), particularly at the early developmental stage P1, with expression levels markedly higher than in narrow leaves (Li and La) or at later stages (P2 and P3) (Figure 6a,b). However, no consistent expression pattern was observed across different tree age stages. Within the DML family, *PeDML1* expression was significantly higher in broad leaves than in narrow leaves, with expression levels gradually declining from P1 to P3, and showing no significant differences between stages A and D. In contrast, *PeDML2* exhibited substantially lower expression levels than *PeDML1* (Figure 6a,b). Furthermore, to validate the accuracy of the RNA-Seq data, four genes (*PeDMT1*, *PeDMT5*, *PeDMT6*, and *PeDML1*) were selected from the 12 Pe*DMT* and *PeDML* genes for qRT-PCR analysis in four samples (Li and Bo in P1 and P3). The qRT-PCR results revealed expression trends consistent with TPM values (Figure 6c), supporting the reliability of the transcriptome data. Collectively, these findings demonstrated that DNA methylation-associated genes play crucial roles in the development of heteromorphic leaves in *P. euphratica*.

### 2.8. The Correlation Between DNA Methylation and Expression of PeDMTs/DMLs During the Development of Heteromorphic Leaves of P. euphratica

The activity of DMTs and DMLs is closely linked to the dynamic changes in DNA methylation. Analysis of DNA methylation data from heteromorphic leaves of *P. euphratica*, previously published by our research group, revealed largely consistent dynamic changes across the three types of DNA methylation (CG, CHG, and CHH). Methylation levels were generally lower in broad leaves compared to narrow leaves and lower in the early P1 stages of leaf development than in the P2 and P3 stages (Figure 6d). In contrast, no clear patterns emerged across different tree ages. Considering the expression patterns of *PeDMT* and *PeDML* genes together (Figure 6b), the low methylation levels observed during early leaf development and in broad leaves are most likely attributable to elevated *PeDML* expression.

## 3. Discussion

In plant genomes, DNA methylation serves as a crucial epigenetic modification mechanism that regulates gene expression, enabling plants to respond to changing environmental conditions and thereby maintain normal growth and developmental processes. This reversible modification is primarily mediated by two functionally antagonistic groups of enzymes: DMTs, which catalyze the addition of methyl groups to cytosine residues, and DMLs, which remove these marks. Together, these enzymes maintain a dynamic balance that is critical for normal plant growth and adaptation. Although DMT and DML gene families have been systematically characterized in model and crop plants such as *A. thaliana* [39], peanuts [25], *Fragaria vesca* [40], *Camellia sinensis* [15], *Dendrobium officinale* [14], and *Ipomoea batatas* L. [26], their identification and functional analysis in desert-adapted tree species have remained limited. This study presents the first systematic identification of DMT and DML gene families in the desert riparian forest species *P. euphratica* and *P. pruinosa*. The identification of these epigenetic regulators not only enhances our understanding of DNA methylation in desert poplars but also lays a foundation for future applied research. Specifically, modulating the expression or activity of key DMT and DML genes may improve plant adaptability to environments.

Comparative genome analysis revealed interspecific asymmetry in the composition of these gene families: *P. euphratica* harbored eight PeDMTs and two PeDMLs, whereas *P. pruinosa* possessed six PpDMTs and three PpDMLs. Such differences may be indicative of lineage-specific gene expansion or loss events following the divergence of these two species. Furthermore, chromosomal distribution analysis suggested that gene localization, while partially conserved between the two species, exhibited species-specific enrichment patterns. These patterns may reflect distinct evolutionary strategies or selection pressures during adaptation to contrasting environmental niches. From the perspective of protein structure, the DMT and DML families exhibit significant functional differentiation characteristics. DMT proteins universally carry a C-5 cytosine methyltransferase domain, forming a stable, compact structure (66–381 aa, 64% of members have an instability index < 40). In contrast, DML proteins contain characteristic permuted zf-CXXC, RRM-fold, and HhH-GPD domains (Figure 3). Their larger MW (862–1876 aa) and high instability (100% of members have an instability index > 40) were more flexible and transient. Subcellular localization prediction revealed that most DMT and all DML proteins were localized in the nucleus, suggesting that these proteins may exert their biological functions within the nucleus, which is consistent with previous studies [15,41,42,43].

Phylogenetic analysis indicates that genes within the same subfamily often share functional similarity. The phylogenetic analysis of the DMT and DML gene families in *A. thaliana*, *P. euphratica*, and *P. prunosa* was conducted (Figure 2). Consistent with findings in *A. thaliana* [44], the DMT genes of both poplar species were classified into four evolutionarily conserved subfamilies: DRM, DNMT, MET, and CMT, indicating strong functional and structural conservation within *Populus*. Conversely, classification of the DML gene family revealed significant species-specific divergence. While *P. prunosa* DML genes grouped into three subfamilies (DME, ROS, and DML), *P. euphratica* lacked a distinct DML subfamily, retaining only DME and ROS. This absence suggested potential lineage-specific evolutionary trajectories or functional diversification within the DML family. The identification of conserved motifs and gene structure patterns within phylogenetically clustered genes further supports functional redundancy and subfamily-specific conservation (Figure 3). Notably, DMT genes demonstrated pronounced variability in gene length and exon number, indicating greater structural plasticity, which may contribute to functional diversification. In contrast, DML genes displayed more conserved gene structures across species and phylogenetic clades. This relative conservation suggested that genes involved in active DNA demethylation may be subject to stronger evolutionary constraints, likely to ensure the stability of their catalytic function [45]. The analysis of cis-acting regulatory elements provided further insights into the potential regulatory roles of these gene families. Consistent with previous studies [15,26], the presence of numerous light-responsive elements (such as the G-box, Box-4, and GT1-motif), hormone-responsive elements (e.g., ABRE), and stress-related elements (e.g., ARE) within the 2 kb upstream regions of DMT and DML genes suggests that their expression may be regulated by both environmental signals and internal signaling pathways. Synteny analysis further elucidated evolutionary dynamics. Highly conserved syntenic relationships were observed for orthologous DMT/DML gene pairs between the two poplar species (Figure 4c), underscoring inter-species conservation. However, intra-species segmental duplication events—notably *PeDMT5*/*PeDMT8* and *PeDML1*/*PeDML2* in *P. euphratica* and *PpDML2*/*PpDML3* in *P. prunosa*—were identified, providing a genetic substrate for neofunctionalization.

Although both *P. euphratica* and *P. pruinosa* have evolved in similar desert riparian environments and display heteromorphic leaf phenotypes (Figure S2a), *P. euphratica* exhibits a wider ecological distribution and stronger tolerance to abiotic stresses. This makes *P. euphratica* not only an ecologically important species in arid ecosystems but also an ideal model for exploring the molecular mechanisms underlying phenotypic plasticity in extreme environments. This study focuses on *P. euphratica*, integrating multi-omics data to investigate the dynamic DNA methylation patterns and regulatory networks of key enzyme genes (*DMTs*/*DMLs*) during heteromorphic leaf development, with the goal of uncovering epigenetic strategies employed by desert plants to adapt to environmental adversity.

The 30 leaf samples in this study were divided into three comparison groups based on their characteristics, including leaf age, leaf shape, and tree age. DNA methylation levels across all three sequence contexts (CG, CHG, and CHH) were found to be lower at P1 than at P2 and P3, as revealed by whole-genome BS-seq (WGBS). Similarly, lower methylation levels were observed in narrow leaves (Li and La) compared to broad leaves (Ov and Bo). By contrast, tree age had no significant effect on methylation patterns. These findings suggested that a greater role may be played by methylation-related gene expression in leaf development and morphological regulation than by age-related factors. Correlating these patterns with gene expression data, it was observed that both *PeDMT* and *PeDML* genes were almost down-regulated from P1 to P3. In particular, *PeDML1* was highly expressed during the early stage of leaf development. Given our previous findings that *PeARF* and *PeWOX* genes play key roles in heteromorphic leaf development [46], the upregulation of *PeDML1* in early-stage leaves may facilitate the expression of such developmental regulators by demethylating their promoter regions, although this hypothesis remains to be validated. This regulatory trend has also been reported in fruit crops such as citrus and tomato, where increased methylation levels are associated with DML downregulation during fruit ripening [19,20,47]. However, the precise regulatory mechanisms underlying these changes in *P. euphratica* remain unclear and warrant further investigation via gene knockout or over-expression. Overall, these insights underscore the crucial role of dynamic DNA methylation in regulating leaf development and morphological plasticity in *P. euphratica*.

## 4. Materials and Methods

### 4.1. Identification of the DMT and DML Gene Families

The DMT and DML gene families were analyzed based on the *P. euphratica* [48] and *P. pruinosa* [31] genomes. HMM profiles corresponding to the DMT domain (PF00145) and DML domain (PF00730, PF15629, and PF15628) were downloaded from the Pfam database via InterPro 105.0 (https://www.ebi.ac.uk/interpro/entry/pfam/#table; accessed on 10 April 2025) and scanned to identify the DMT and DML proteins. HMMER (v3.3.2; http://hmmer.org/, accessed on 12 April 2025) was used to search the entire *P. euphratica* and *P. pruinosa* protein sequence for proteins containing DMT and DML domains (E < 0.001). The obtained protein sequences were utilized to construct a new HMM model, which was then applied to conduct a second search on all protein sequences (E < 0.001). Moreover, proteins of the *A. thaliana* DMT and DML gene families were also downloaded from TAIR (https://www.arabidopsis.org/; accessed on 2 April 2025) and subjected to BLASTp (2.5.0+) alignment with the *P. euphratica* and *P. pruinosa* protein sequences (E < 1 × 10^−5^). To ensure reliability, the results from the secondary search and BLASTp analysis were validated using three domain databases: SMART (http://smart.embl-heidelberg.de/; accessed on 11 April 2025), InterPro (https://www.ebi.ac.uk/interpro/; accessed on 11 April 2025), and NCBI-CDD (https://www.ncbi.nlm.nih.gov/cdd/; accessed on 11 April 2025). The physiochemical properties of DMT and DML proteins, including the amino acid length, MW, pI, instability index, and GRAVY, were examined by Expasy [49] (https://web.expasy.org/protparam/, accessed on 12 April 2025). The MG2C [50] online tool (http://mg2c.iask.in/mg2c_v2.0/, accessed on 14 April 2025) was utilized for chromosome localization visualization.

### 4.2. Protein Tertiary Structure Prediction and Subcellular Localization Analysis

The SPOMA [51] online tool (https://npsa.lyon.inserm.fr/cgi-bin/npsa_automat.pl?page=/NPSA/npsa_sopma.html, accessed on 16 April 2025) and SWISS-MODEL [52] online tool (https://swissmodel.expasy.org/, accessed on 16 April 2025) were employed to predict the secondary and tertiary structures of the DMT and DML proteins from *P. euphratica* and *P. pruinosa*, respectively. The DeepTMHMM [53] online tool (https://dtu.biolib.com/DeepTMHMM, accessed on 17 April 2025) was utilized for predicting the transmembrane protein structure of the DMT and DML proteins. Additionally, the WOLF PSORT online tool (https://wolfpsort.hgc.jp/, accessed on 17 April 2025) was applied to predict the subcellular localization of these proteins.

### 4.3. Construction and Analysis of the Evolutionary Tree

Neighbor-Joining phylogenetic trees were constructed using MEGA12 (v12.0.11). The analysis employed MUSCLE-aligned protein sequences from *P. euphratica*, *P. pruinosa*, and *A. thaliana* with 1000 bootstrap replicates. The resulting tree was visualized using the iTOL v7 online tool [54] (https://itol.embl.de/, accessed on 22 April 2025).

### 4.4. Analysis of Gene Structure and Conserved Motif

The MEME [55] program (version 5.5.8) online tool (https://meme-suite.org/meme/tools/meme, accessed on 2 May 2025) was employed to identify conserved motifs present within DMT and DML proteins. Subsequently, the coding sequence (CDS) information of the DMT and DML gene families from the *P. euphratica* and *P. pruinosa* genomes was extracted by TBtools-II (v2.308) [56]. Finally, the identified motif structures, gene structures, and evolutionary relationships were visualized using the TBtools-II (v2.308) software.

### 4.5. Analysis of Cis-Acting Elements and Collinearity

The CDS sequences of the DMT and DML genes located 2kb upstream in *P. euphratica* and *P. pruniosa* were extracted using TBtools-II (v2.308). Subsequently, the cis-acting regulatory elements within these genes were analyzed and visualized using the PlantCARE [57] (https://bioinformatics.psb.ugent.be/webtools/plantcare/html/, accessed on 5 May 2025) online tool and TBtools-II (v2.308), respectively. To identify gene duplication patterns and perform collinearity analysis, TBtools-One Step MCScanX, TBtools-Dual Systeny Plot, and TBtools-Advanced Circos were utilized.

### 4.6. Analysis of RNA-Seq and WGBS Data

The transcriptome and whole-genome methylation data used in this research were derived from our research group’s previously published research on heteromorphic leaf development in *P. euphratica* [29,46], with raw data deposited in the National Genomics Data Center (https://ngdc.cncb.ac.cn/; project number: PRJCA005959). For each leaf type and developmental stage, three biological replicates were included. Heteromorphic leaves—classified as Li, La, Ov, and Bo—were sampled during March and April across four tree developmental stages (A–D) and three leaf developmental stages (P1–P3; Figure S2b). Gene expression quantification was performed using TPM values generated by StringTie v2.1.7 [58], while methylation sites were identified using Bismark (v0.22.3) [59] software.

### 4.7. qRT-PCR Validation

The qRT-PCR experiments were conducted on the mRNA from two *P. euphratica* heteromorphic leaves (Li and Bo) in P1 and P3 of the D stage. Primers used are listed in Table S5. Reactions were run in triplicate on an ABI 7500 real-time PCR system (Thermo Fisher Scientific, Waltham, MA, USA), using the *PeActin* sequence as the endogenous control. The CT values were analyzed using the 2^−ΔΔCT^ method [60].

## Data Availability

Sequence data from this article can be found in the National Genomics Data Center (https://ngdc.cncb.ac.cn/) with the project number PRJCA005959.

## Supplementary Materials

The following supporting information can be downloaded at https://www.mdpi.com/article/10.3390/plants14152370/s1: Figure S1: Predicted tertiary structures of certain DMT family members in *P. euphratica* and *P. pruinosa*; Figure S2: Morphological characteristics of heteromorphic leaves. (a) Photographs of tree and leaf shape in *P. euphratica* and *P. pruinosa*. (b) Different leaf shapes in *P. euphratica* of four tree stages at three development leaf stages. Tree stages: A Stage (3 year): Li; diameter: 2.3 cm, height: 4.1 m; B Stage (5 year): Li and La; diameter: 4.0 cm, height: 4.6 m; C Stage (7 year): Li, La, and Ov; diameter: 5.1 cm, height: 5.1 m; D Stage (8 year): Li, La, Ov, and Bo; diameter: 8.2 cm, height: 7.2 m. Leaf stages: P1: complete leaf flattening (initial phase); P2: intermediate stage (day 15); P3: maturation phase (day 30); Table S1: Molecular characteristics of DMT and DML gene families across *P. euphratica* and *P. pruinosa*; Table S2: Prediction of secondary structure of DMT and DML proteins in *P. euphratica* and *P. pruinosa*; Table S3: Prediction of subcellular localization of DMT and DML proteins in *P. euphratica* and *P. pruinosa*; Table S4: The conserved motif of DMT and DML; Table S5: The primers of genes in the qRT-PCR experiment.

## Abbreviations

The following abbreviations are used in this manuscript:

DMT	DNA methyltransferase
DML	DNA demethylase
MET	methyltransferase
CMT	chromomethylase
DRM	domains rearranged methylase
DNMT2	DNA methyltransferase 2
ROS1	repressor of silence 1
DME	transcriptional activator demeter
DML2	demeter-like protein 2
DML3	demeter-like protein 3
HMM	Hidden Markov Model
GRAVY	grand average of hydrophobicity
Li	linear
La	lanceolate
Ov	ovate
Bo	broad-ovate
TPM	transcripts per million
MW	molecular weight
pI	isoelectric point
CDS	coding sequence
