# Supplementary material for: Genome-Wide Identification of DNA Methyltransferase and Demethylase in Populus sect. Turanga and Their Potential Roles in Heteromorphic Leaf Development in Populus euphratica
Source: Plants (Basel). 2025 Aug 1;14(15):2370. doi: 10.3390/plants14152370 (PMC12349049; doi:10.3390/plants14152370)
Supplement: Supplementary file 1 [file plants-14-02370-s001.zip › Supplementary Figures.pdf]

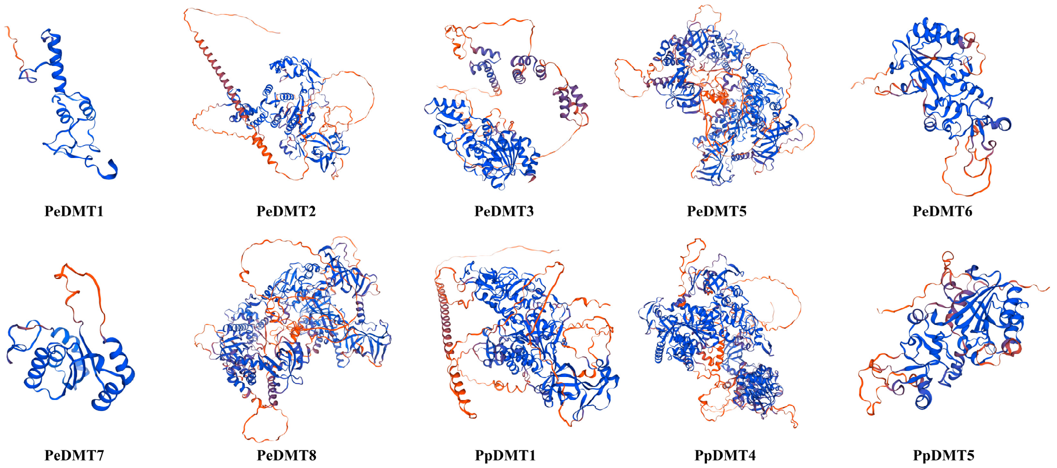

Figure S1. Predicted tertiary structures of certain DMT family members in *P. euphratica* and *P. pruinosa*.

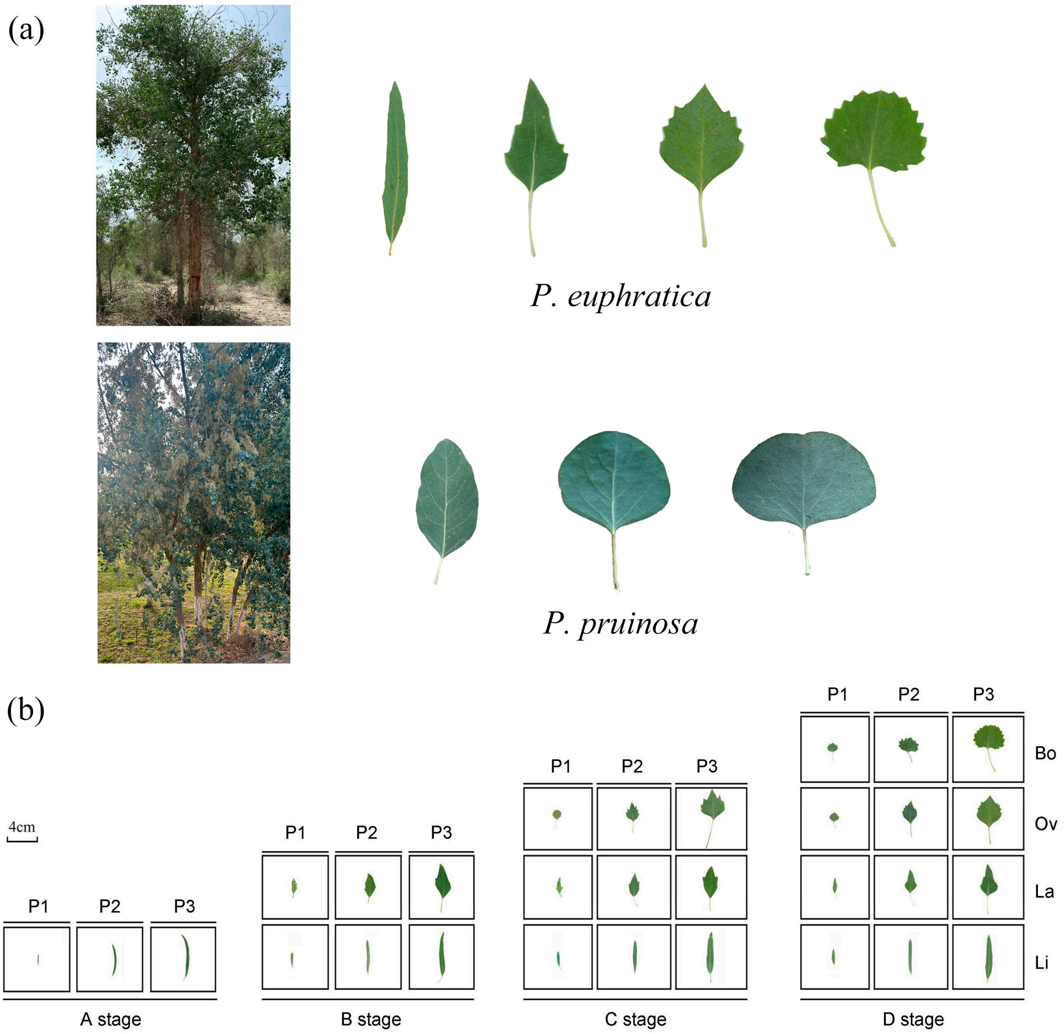

Figure S2. Morphological characteristics of heteromorphic leaves. (a) Photographs of tree and leaf shape in *P. euphratica* and *P. pruinosa*. (b) Different leaf shapes in *P. euphratica* of four tree stages at three development leaf stages. Tree stages: A Stage (3 year): Li; diameter: 2.3 cm, height:

4.1 m; B Stage (5 year): Li and La; diameter: 4.0 cm, height: 4.6 m; C Stage (7 year): Li, La, and Ov; diameter: 5.1 cm, height: 5.1 m; D Stage (8 year): Li, La, Ov, and Bo; diameter: 8.2 cm, height: 7.2 m. Leaf stages: P1: complete leaf flattening (initial phase); P2: intermediate stage (day 15); P3: maturation phase (day 30).
